# Prediction of papillary thyroid metastases to the central compartment: proposal of a model taking into consideration other thyroid conditions

**DOI:** 10.3389/fendo.2023.1299290

**Published:** 2023-11-28

**Authors:** Qiong Chen, Xiaofen Ye, Kangjian Wang, Haolin Shen

**Affiliations:** ^1^ Department of Ultrasound, Zhangzhou Affiliated Hospital of Fujian Medical University, Zhangzhou, Fujian, China; ^2^ School of Clinical Medicine, Fujian Medical University, Fuzhou, Fujian, China

**Keywords:** central lymph node metastasis, papillary thyroid carcinoma, ultrasonography, thyroid, disease

## Abstract

**Objective:**

To construct risk prediction models for cervical lymph node metastasis (CLNM) of papillary thyroid carcinoma (PTC) under different thyroid disease backgrounds and to analyze and compare risk factors among different groups.

**Methods:**

This retrospective study included 518 patients with PTC that was pathologically confirmed post-operatively from January 2021 to November 2021. Demographic, ultrasound and pathological data were recorded. Univariate and multivariate logistic regression analyses were performed to identify factors associated with CLNM in the whole patient cohort and in patients grouped according to diagnoses of Hashimoto’s thyroiditis (HT), nodular goiter (NG), and no background disease. Prediction models were constructed for each group, and their performances were compared.

**Results:**

Analysis of the whole PTC patient cohort identified NG as independently associated with CLNM. The independent risk factors for patients with no background disease were the maximum thyroid nodule diameter and American College of Radiology Thyroid Imaging Reporting & Data System score; those for patients with HT were the maximum thyroid nodule diameter, ACR TI-RADS score, and multifocality; and those for patients with NG were the maximum thyroid nodule diameter, ACR TI-RADS score, multifocality and gender.

**Conclusion:**

Background thyroid disease impacts CLNM in PTC patients, and risk factors for CLNM vary among PTC patients with different background diseases. Ultrasound is useful for diagnosing background thyroid disease, which can inform treatment planning. Different prediction models are recommended for PTC cases with different thyroid diseases.

## Introduction

Thyroid carcinoma has had the fastest rising incidence among all carcinomas over the past decade. Papillary thyroid carcinoma (PTC) is the predominant type of thyroid carcinoma, accounting for 84%–99% of all cases of thyroid cancer (1, 2). Patients with PTC have a better prognosis when the lesion is confined to the gland (3). The risk of recurrence is increased (4), and PTC patients’ prognosis is poor once central lymph node metastasis (CLNM) has occurred. If metastatic lymph nodes are detected before surgery, patients should receive prophylactic central compartment neck dissection (pCCND) to prevent the recurrence and further metastasis of PTC. Therefore, methods to accurately determine pre-operatively whether CLNM has occurred in PTC patients and strategies to adapt corresponding treatment plans represent a major clinical need.

The fine structure of tissues can be visualized via ultrasound imaging, which is the technique of choice for detecting thyroid lesions and evaluating CLNM (5–7). However, direct preoperative identification of CLNM remains a challenge. Only approximately 1/3 of PTC patients with CLNM have detectable involved nodes at the time of presentation (8). Clinicians are often inclined to perform lymph node dissection at the same time as thyroidectomy to avoid missing lymph node metastasis (LNM). However, thyroidectomy accompanied by CLN dissection is associated with increased risks of permanent hypoparathyroidism and unintentional, permanent laryngeal nerve injury (9). Patients with very low risk of metastasis are not likely to benefit from pCCND (10). Therefore, a detailed assessment of the CLN status before surgery to avoid unnecessary pCCND is essential.

Many studies have explored risk factors related to CLNM and constructed risk prediction models. The study by Mao et al. (11) identified age (<45 years old), gender (male), multifocality, tumor size (>1 cm), and thyroid extracapsular invasion as factors that increase the risk of lymph node metastasis in PTC patients. Shen et al. (12) identified gender (male), multifocality, maximum lesion diameter, and American College of Radiology Thyroid Imaging Reporting & Data System (ACR TI-RADS) score as independent risk factors for CLNM. Kim et al. (13) concluded that Hashimoto’s thyroiditis (HT) and PTC tend to occur at the same time and HT is known to increase the risk of PTC, but HT may also attenuate tumor aggressiveness and metastasis. However, Yang et al. (14) believed that the risk of CLNM is greater in PTC patients with nodular goiter (NG). To our knowledge, no studies have yet compared and analyzed risk factors for CLNM in PTC patients with HT or NG.

Therefore, the present study had the following objectives: 1. to verify the correlation between background thyroid disease (HT and NG) and CLNM in PTC patients; 2. to explore potential differences in risk factors between PTC patients with HT and PTC patients with NG; and 3. to construct prediction models for CLNM in PTC patients with different background thyroid diseases and compare their diagnostic performances.

## Materials and methods

### Patients

For this retrospective study, we analyzed 665 patients with pathologically proven PTC who underwent partial or total thyroidectomy in our hospital between January 2021 and December 2021. All patients underwent lymph node dissection. Patients with unilateral thyroid nodules received ipsilateral neck lymph node dissection, and those with bilateral thyroid nodules underwent bilateral neck dissection. The following clinical information was recorded for each patient: age, gender, history of thyroid disease, surgical details, and pathological results.

The inclusion criteria for PTC patients were as follows (1): age >18 years; and (2) complete medical records: including detailed medical history, preoperative ultrasound examination of thyroid and cervical lymph nodes, and pathological results. For cases with multiple PTC nodules, the nodule with the highest ACR TI-RADS score was selected. If the ACR TI-RADS scores were the same for multiple PTC nodules, the nodule with the maximum diameter was selected for analysis. The exclusion criteria were as follows (1): previous history of thyroidectomy or head-and-neck radiation therapy (2), The nodules assessed and recorded by ultrasound were inconsistent with the pathologically confirmed lesions (3), uncertain pathological results (4), incomplete US imaging data and images (5), bilateral lobe PTC with CLNM on only one side (6), pathological results showing metastatic lymph nodes in only the lateral cervical region (7), suspicious metastatic lymph nodes on the neck that can be found by ultrasound, removing the need for prediction, and (8) presence of both HT and NG.

According to the pathological results, the patients were divided into three groups of those without thyroid disease, those with HT, and those with NG. The inclusion conditions for HT (1): ultrasonography showed the typical appearance of HT, which includes reduced echogenicity, with or without thyroid nodules (2); consistent with the diagnosis criteria for HT (15), including increased titers of TPOAb and TgAb; or (3) confirmed by biopsy or surgical pathology. The inclusion conditions for NG (1): diffuse parenchymal thyroid lesions with or without nodules on ultrasonography (2); laboratory examination of normal thyroid function, normal titers of TPOAb and TgAb, and confirmed by biopsy or surgical pathology. The patients in each group were further divided into groups with and without metastasis.

The ethics committee of our hospital approved this retrospective study (Protocol No. 2022KYB138). All procedures performed in studies involving human participants were in accordance with the ethical standards of the institutional and/or national research committee and with the 1964 Helsinki declaration and its later amendments or comparable ethical standards. All included patients provided written informed consent.

### Ultrasound examination

All images were acquired using an Acuson S3000 (Siemens Medical Solutions USA, Malvern, PA) equipped with a 7- to 9-MHz linear-array transducer, a Hitachi vision Preirus (Hitachi Medical Crop., Chiba, Japan) equipped with a 7- to 13-MHz linear-array transducer, or a Mindray Resona 7S (Mindray BioMedical, Shenzhen, China) equipped with a 7- to 14-MHz linear-array transducer.

Based on the ACR TI-RADS score, thyroid lesions were analyzed by a physician with 22 years of ultrasound experience who was blinded to the pathological results. The following ultrasound features of lesions were recorded: number, location, size(maximum diameter), composition, echogenicity, shape, margin and echogenic foci.

### Statistical analysis

Statistical analyses were performed using R version 4.2.1. The Kolmogorov–Smirnov test was used to analyze the distribution of continuous variables. The results show that all continuous variables followed abnormal distributions, and thus, the data are presented as median [interquartile range]. The *rms* package was used to perform univariate and multivariate analyses and to construct predictive models. Each feature with a Wald test *P* value<0.25 in the univariate analysis was included in a multivariate logistic regression model to estimate the corresponding adjusted odds ratio (OR) and corresponding 95% confidence interval (CI). Final model selection was performed by a backward step-down selection process using the Akaike information criterion (AIC). The *val. prob ()* function was used to calculate the C-index and draw the calibration curve. The bootstrap sampling method was used to evaluate the regression coefficient. *P* values<0.05 indicated statistical significance.

## Results

### Characteristics of PTC nodules

**Figure 1:**
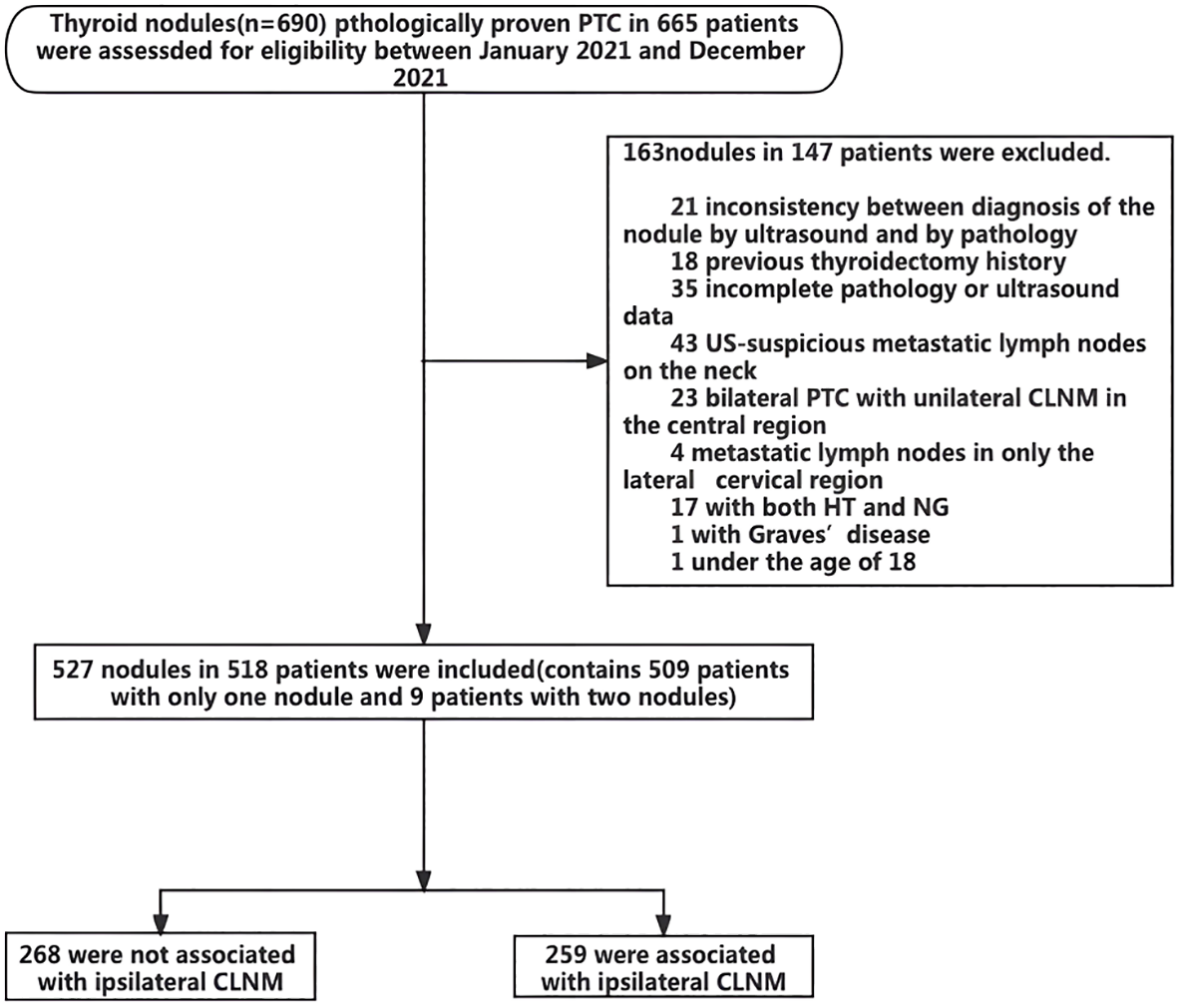
Flow chart of selection of PTC patients and nodules. n = number of nodules.

**Figure 1** presents a flow chart of the selection of PTC patients and nodules for analysis in the present study. Overall, 149 patients with 165 nodules were excluded due to the indicated causes. Finally, a total of 518 patients with 527 nodules were included. Of these patients, 509 patients had only one PTC nodule, and 9 patients had two PTC nodules. Among the 527 analyzed PTC nodules, 268 were not associated with ipsilateral CLNM, and 259 were associated with ipsilateral CLNM. The patients ranged in age from 18 to 80 years and included 411 male patients and 116 female patients. Multifocality was observed in 362 patients. Overall, 112 patients had HT, and 283 patients had NG. The maximum nodule diameter of thyroid nodules ranged from 1 to 57 mm, and the ACR score ranged from 4 to 14 points (**Table 1**).

**Table 1:** Demographic and ultrasound characteristics of PTC patients with or without CLNM.

Characteristics	Total	Without CLNM (%) n=268 nodules	With CLNM (%) n=259 nodules
Age, y*	47[39, 55]	48 [40, 54]	46 [37, 56]
Gender, n (%)			
Female	411(77.9)	227 (55.2)	184 (44.7)
Male	116(22.1)	41 (35.5)	75 (64.5)
Multifocality, n (%)			
No	362(68.6)	197 (54.4)	165 (45.6)
Yes	165(31.4)	71 (43.0)	94 (57.0)
Thyroid background disease, n (%)			
None	132(25.0)	73 (55.3)	59 (44.6)
Hashimoto’s thyroiditis	112(21.2)	69 (61.6)	43 (38.4)
Nodular goiter	283(53.7)	126 (44.5)	157(55.4)
Maximum nodule diameter, mm*	8[5,11]	6 [5,9]	10 [6.4,15]
ACR score (points)*	10[9,11]	9.0 [7,10]	10 [9,12]

*Median [interquartile range].

### Risk factor analysis in whole PTC patient cohort

The results of univariate and multivariate logistic analyses identified NG as independently associated with CLNM in all included PTC patients (*OR*=1.7, *P*=0.027), as well as gender, multifocality, maximum nodule diameter, and ACR TI-RADS score (**Table 2**). The risk of CLNM was higher for male patients vs. female patients and for cases with multifocality vs. those without. Additionally, the risk of CLNM increased with each 1-mm increase in PTC nodule diameter and with increasing ACR TI-RADS score.

**Table 2:** Univariate and multivariate analyses of risk factors related to CLNM in the whole PTC patient cohort.

	Univariate analysis		Multivariate analysis	
	*OR* (95%*CI*)	*P* value	*OR* (95%*CI*)	*P* value
Age	0.99 (0.98, 1.01)	0.382		
Gender				
Female	reference	–	reference	–
Male	2.26 (1.47, 3.46)	<0.001	1.84 (1.13, 2.97)	0.013
Multifocality	1.58 (1.09, 2.29)	0.015	1.78 (1.15, 2.75)	0.009
Thyroid background disease		0.003		0.006
None	reference	–	reference	–
Hashimoto’s thyroiditis	0.77 (0.42, 1.29)	0.320	0.81 (0.45, 1.44)	0.474
Nodular goiter	1.54 (1.02, 2.34)	0.041	1.7 (1.06, 2.76)	0.027
Maximum nodule diameter	1.15 (1.10, 1.19)	<0.001	1.14 (1.09, 1.18)	<0.001
ACR TI-RADS score	1.25 (1.15, 1.35)	<0.001	1.28 (1.17, 1.40)	<0.001

### Risk factors analysis and model construction in PTC patients without background thyroid disease

The independent risk factors for CLNM in PTC patients without background thyroid disease were found to be the maximum nodule diameter and ACR TI-RADS score (**Table 3**).

**Table 3:** Univariate and multivariate analyses of risk factors related to CLNM in PTC patients without background thyroid disease.

	with CLNM	without CLNM	Univariate analysis		Multivariate analysis	
			*OR (95%CI)*	*P* value	*OR (95%CI)*	*P* value
Age	47 [34, 57]	46 [40.5, 53]	0.99 (0.95,1.03)	0.765		
Gender						
Female	40 (41.7)	56 (58.3)	reference	–		
Male	19 (52.7)	17 (47.2)	1.56 (0.72,3.38)	0.254		
Multifocality	50 (37.8)	82 (62.1)	0.74 (0.36,1.50)	0.396		
Maximum nodule diameter	10.2 [6.3,16.6]	5.6 [4,8]	1.22 (1.12,1.33)	<0.001	1.24 (1.31,1.36)	<0.001
ACR TI-RADS score	10 [9,11]	9 [7,10]	1.24 (1.05,1.47)	0.008	1.39 (1.13,1.70)	<0.001

According to the lowest AIC value (140.27), model_1 for CLNM prediction in these patients was constructed as follows:


Y=−3.86 + 0.23×X1 + 0.33×X2 − 0.04×X3

where X1 is the maximum nodule diameter (mm), X2 is the ACR TI-RIDS score, and X3 is the patient’s age. The 95% *CIs* for the regression coefficients for maximum nodule diameter, ACR TI-RIDS score, and age were 0.14–0.34, 0.08–0.62, and -0.07–0.002, respectively.

**Figure 2:**
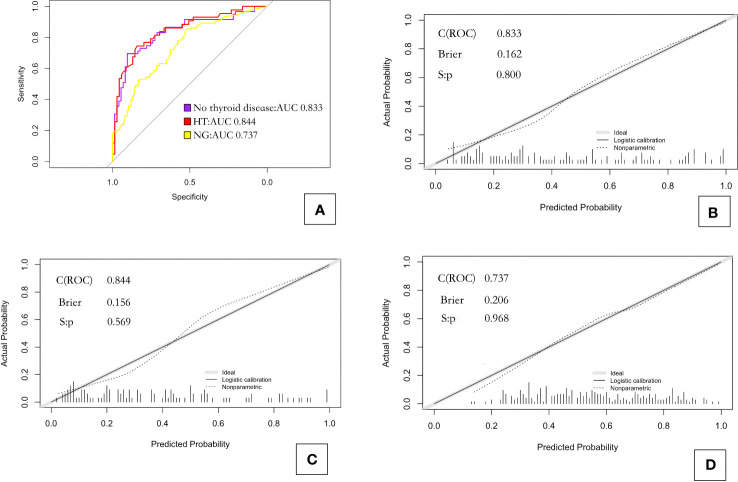
ROC curves and calibration curves for the three prediction models. **(A)** ROC curves for the three models; **(B)** calibration curve for model_1 predicting CLNM in PTC patients without background thyroid disease; **(C)** calibration curve for model_2 predicting CLNM in PTC patients with Hashimoto’s thyroiditis; and **(D)** calibration curve for model_3 predicting CLNM in PTC patients with nodular goiter. HT, Hashimoto’s thyroiditis; NG, nodular goiter.

Receiver operating characteristic (ROC) curve analysis showed that 0.493 was the best diagnostic cutoff value (**Figure 2A**). The C-index of model_1 was 0.833; the Brier index was 0.162, and the *P* value from the unreliability test was 0.800 (**Figure 2B**).

### Risk factors analysis and model construction in PTC patients with HT

The independent risk factors for CLNM in PTC patients with HT were multifocality, maximum nodule diameter, and ACR TI-RADS score (**Table 4**).

**Table 4:** Univariate and multivariate analyses of risk factors related to CLNM in PTC patients with Hashimoto’s thyroiditis.

	with CLNM	without CLNM	Univariate analysis		Multivariate analysis	
			*OR (95%CI)*	*P* value	*OR (95%CI)*	*P* value
Age	43 [37, 55]	47 [39, 53]	0.99 (0.96,1.02)	0.508		
Gender						
Female	39 (37.5)	62 (62.5)	reference	–		
Male	4(50.0)	4 (50.0)	1.67 (0.39,7.05)	0.489		
Multifocality	49 (43.7)	63 (56.2)	1.9 (0.88,4.12)	0.101	2.85 (1.1,7.42)	0.027
Maximum nodule diameter	11 [7, 16]	6 [5, 9]	1.28 (1.14,1.44)	<0.001	1.30 (1.14,1.48)	<0.001
ACR TI-RADS score	10 [9, 13]	9 [9, 10]	1.29 (1.07,1.55)	0.005	1.37 (1.09,1.71)	0.004

According to the lowest AIC value (119.87), model_2 for CLNM prediction in these patients was constructed as follows:


Y=−6.47 +0.26×X1 + 0.31×X2 + 1.04×X3

where X1 is the maximum nodule diameter (mm), X2 is the ACR TI-RIDS score, and X3 is multifocality (absence=0, presence=1). The 95% *CIs* for the regression coefficients for maximum nodule diameter, ACR TI-RIDS score, and multifocality were 0.05–0.42, 0.03–0.60, and 0.15–2.27, respectively.

ROC curve analysis showed that 0.439 was the best diagnostic cutoff value (**Figure 2A**). The C-index of model_2 was 0.844; the Brier index was 0.156; and the *P* value from the unreliability test was 0.569 (**Figure 2C**).

### Risk factors analysis and model construction in PTC patients with NG

The independent risk factors for CLNM in PTC patients with NG were gender, multifocality, maximum nodule diameter, and ACR TI-RADS score (**Table 5**).

**Table 5:** Univariate and multivariate analyses of risk factors related to CLNM in PTC patients with nodular goiter.

	with CLNM	without CLNM	Univariate analysis		Multivariate analysis	
			*OR (95%CI)*	*P* value	*OR (95%CI)*	*P* value
Age	47 [37.5, 55.5]	48 [41, 55]	0.99 (0.97,1.01)	0.364		
Gender						
Female	105 (49.8)	106 (50.2)	reference	–	reference	–
Male	4(9.3)	4 (5.7)	2.62 (1.47,4.70)	<0.001	2.39 (1.28,4.48)	0.005
Multifocality	66(23.3)	217(76.6)	3.56 (1.89,6.71)	<0.001	2.73 (1.4,5.35)	0.002
Maximum nodule diameter	9.5 [6, 13.5]	7 [5, 10]	1.09 (1.04,1.14)	<0.001	1.07 (1.03,1.12)	<0.001
ACR TI-RADS score	10 [9, 12]	9 [7, 10]	1.24 (1.11,1.39)	<0.001	1.24 (1.10,1.39)	<0.001

According to the lowest AIC value (347.18), model_3 for CLNM prediction in these patients was constructed as follows:


Y=−2.95 +0.07×X1 + 0.21×X2 + 1.01×X3 + 0.87×X4

where X1 is the maximum nodule diameter (mm), X2 is the ACR TI-RIDS score, X3 is multifocality (absence=0, presence=1), and X4 is gender (0=female, 1=male). The 95% *CIs* for the regression coefficients for maximum nodule diameter, ACR TI-RIDS score, multifocality, and gender were 0.03–0.12, 0.08–0.33, 0.26–1.70, and 0.16–1.55, respectively.

ROC curve analysis showed that 0.604 was the best diagnostic cutoff value (**Figure 2A**). The C-index of the model_3 was 0.737; the Brier index was 0.206; and the *P* value from the unreliability test was 0.968 (**Figure 2D**).

## Discussion

Knowledge of whether CLNM has occurred is critical to determining the extent of neck dissection for PTC patients. The present study revealed that background thyroid disease is independently related to the occurrence of CLNM. The prevalence of HT in Aisa is 5.8% (16), previous research has indicated that HT may affect recurrence and overall survival among PTC patients due to tumor cell killing via the autoimmune response as well as a lower frequency of mutations associated with more aggressive disease (17). Our results also showed that HT did not increase the risk of CLNM in PTC patients (*OR*=0.77, *P*=0.320). However, our analyses indicated that PTC patients with NG were more likely to experience CLNM (*OR*=1.54, *P*=0.041). Metastasis is commonly attributed to high thyroid-stimulating hormone concentrations in PTC patients with NG (18). According to our findings, NG should be taken into consideration when evaluating the likelihood of CLNM in PTC patients. The results of this study also indicated that among all PTC patients, male gender, large nodule diameter, multifocality, high ACR TI-RADS score, and background thyroid disease can be used as independent risk predictors, which is consistent with the findings of Shen et al. (11) and Yang et al. (14). Another previous study also considered age<45 years as a relevant risk factor for PTC patients with HT, but our study did not find a clear association between these factors (19).

To further study the influence of background thyroid disease on CLNM, we performed a stratified analysis of patients divided into three groups, according to the absence of background thyroid disease, presence of HT, and presence of NG. The results suggested that the maximum diameter of nodules and ACR TI-RADS score were independent risk factors in all three groups. The risk of CLNM in the central region increased with each 1-mm increase in PTC nodule diameter, which is consistent with previous research indicating that tumor size is a predictor of CLNM even for PTC diagnosed without clinical node metastases (20). Our analysis results also indicated that the risk of CLNM increased with increasing ACR TI-RADS score in all groups.

Some differences in the independent risk factors for CLNM were observed between PTC patients with HT and NG. Multifocality was not an independent risk factor for CLNM in PTC patients without background thyroid disease, but was an independent risk factor in PTC patients with HT and NG, which is consistent with previous studies (21, 22). This finding suggests that when fine-needle aspiration of the thyroid gland is performed, puncture of each suspicious nodule facilitates a more accurate preoperative assessment. Gender was not associated with CLNM in PTC patients with HT but was in PTC patients with NG, and male patients seemed to have a higher risk of CLNM than female patients (*OR=*2.37, *P*=0.005), which agrees with the findings of a previous study (23).

Because the independent risk factors for CLNM in PTC patients varied among the groups, we established three different prediction models based on the AIC values and tested them by the bootstrap principle. ROC curve analyses provided AUC values for the different models, and these values were 0.836, 0.844, and 0.738 for the models constructed for PTC patients without background thyroid disease, with HT, and with NG, respectively. These results showed that each model had good discriminatory performance for predicting CLNM. The Brier indexes for the different models obtained via calibration curve analyses were 0.162, 0.156, and 0.206, respectively. These values indicate that the agreement between the predicted and observed probability of CLNM was good for each model, and thus, the models offer good clinical reliability. Based on the large-scale application of thyroid fine-needle aspiration biopsy in clinical practice, we recommend adding the pathological results as a reference, focusing on whether PTC patients have background thyroid disease, and selecting the corresponding prediction model according to the type of disease. This approach may provide more effective evaluation of whether CLNM has occurred in the central region of PTC patients and may benefit clinicians in formulating surgical plans.

The present study has some limitations. The group of patients with HT included only eight male patients. Thus, the significance of gender as an independent risk factor for predicting CLNM in this group must be further verified in a larger study. Meanwhile, another type of thyroid disease (Graves’ disease) could not be included in the present analysis due to the small number of patients with this disease. Previous research has suggested that factors including age, gender, overweight, and place of residence may affect the occurrence of PTC and LNM in patients with NG (24), and several of these factors were not analyzed in this study. Such analyses will also be included in our future research. Such analyses will also be included in our future research. Fourth, more prospective studies are needed to validate the efficacy of the prediction models.

## Conclusion

Nodular goiter has an impact on CLNM in PTC patients, and risk factors for CLNM differ among PTC patients with different background thyroid diseases. Therefore, different prediction models are recommended for assessing the risk of CLNM in PTC patients with different thyroid diseases. Finally, ultrasound is useful for the diagnosis of background thyroid disease, and the results can be applied in prediction models to help clinicians in treatment planning.

## Data availability statement

The original contributions presented in the study are included in the article/supplementary material. Further inquiries can be directed to the corresponding author.

## Ethics statement

This retrospective study was approved by the research ethics committee of Zhangzhou Affiliated Hospital of Fujian Medical University (Protocol No. 2022KYB138). All procedures performed in studies involving human participants were in accordance with the ethical standards of the institutional and/or national research committee and with the 1964 Helsinki declaration and its later amendments or comparable ethical standards. The studies were conducted in accordance with the local legislation and institutional requirements. Informed consent was obtained from all individual participants included in the study.

## Author contributions

QC: Writing – original draft, Conceptualization, Project administration, Supervision, Visualization. XY: Methodology, Resources, Writing – review & editing. KW: Project administration, Visualization, Writing – original draft. HS: Investigation, Project administration, Supervision, Visualization, Writing – original draft, Writing – review & editing.
